# Upper Extremity Hematoma: An Atypical Case of Compartment Syndrome

**DOI:** 10.7759/cureus.12453

**Published:** 2021-01-03

**Authors:** Nicholas Cowell, Andrew Kalnow

**Affiliations:** 1 Emergency Medicine, OhioHealth Doctors Hospital, Columbus, USA; 2 Emergency Medicine, Ohio University Heritage College of Osteopathic Medicine, Athens, USA

**Keywords:** compartment syndrome, atypical presentation, emergency medicine

## Abstract

Compartment syndrome is a potentially life- or limb-threatening condition that carries high morbidity if not promptly diagnosed and treated. This case is an atypical presentation demonstrating why a high index of suspicion, prompt surgical consultation, and intervention is required if compartment syndrome is suspected.

## Introduction

Compartment syndrome occurs when compartment pressures within an enclosed facial space exceed venous capillary pressures [1-3]. As pressures rise within a space enclosed by fascia, worsening pain, numbness, weakness, and, eventually, pulselessness can develop. The most sensitive physical exam finding is pain with passive stretching [1]. Distal pulses typically remain unaffected early in the development of compartment syndrome [2]. While tissue pressures may help make the diagnosis of compartment syndrome, the exam alone should be sufficient. As pressures within the affected compartments rise, worsening pain followed by numbness in the nerves traversing the compartment can develop [1]. Prompt surgical consultation and intervention is necessary, as irreversible damage can occur in as little as six hours [1]. Trauma remains the leading cause of compartment syndrome with the majority occurring in the lower extremities [1-2]. Here, we present a rare case of compartment syndrome with an atypical presentation highlighting the importance of serial examinations and a high index of suspicion.

## Case presentation

A 71-year-old male with a history of stage IV lung cancer status post radiation and palliative chemotherapy completed two months ago presented to the emergency department (ED) approximately 20 minutes after an episode of syncope while standing. The patient stated he became dizzy prior to the fall and now complains of pain in his scalp. The patient denied the use of anticoagulant or antiplatelet medications. At the time of presentation to the ED, he denied active dizziness, nausea, vomiting, chest pain, or shortness of breath. Physical exam revealed an alert, oriented, age-appropriate male in no distress. His initial exam, including a complete neurologic assessment, was unremarkable, with the exception of a 3 cm laceration to his scalp, a 1 cm laceration to his occiput, and a small skin tear to his right lateral elbow with mild, soft tissue swelling. Bleeding was well-controlled upon arrival to the ED from all laceration sites with dressings applied by first responders.

The right elbow to which he sustained the skin tear had minimal tenderness to palpation on the dorsal aspect around the lesion with minimal swelling but no additional deformity or crepitus. The patient had intact pulse and motor and sensory function with a full range of motion of the right elbow, shoulder, and wrist without pain.

Initial workup included computed tomography (CT) head without contrast, complete blood count (CBC), comprehensive metabolic panel (CMP), urinalysis (UA), electrocardiogram (EKG), and troponin. Laboratory analysis and imaging were unremarkable and at this point in the patient course, the working diagnosis was syncope and collapse with a closed head injury and associated uncomplicated lacerations.

**Figure 1:**
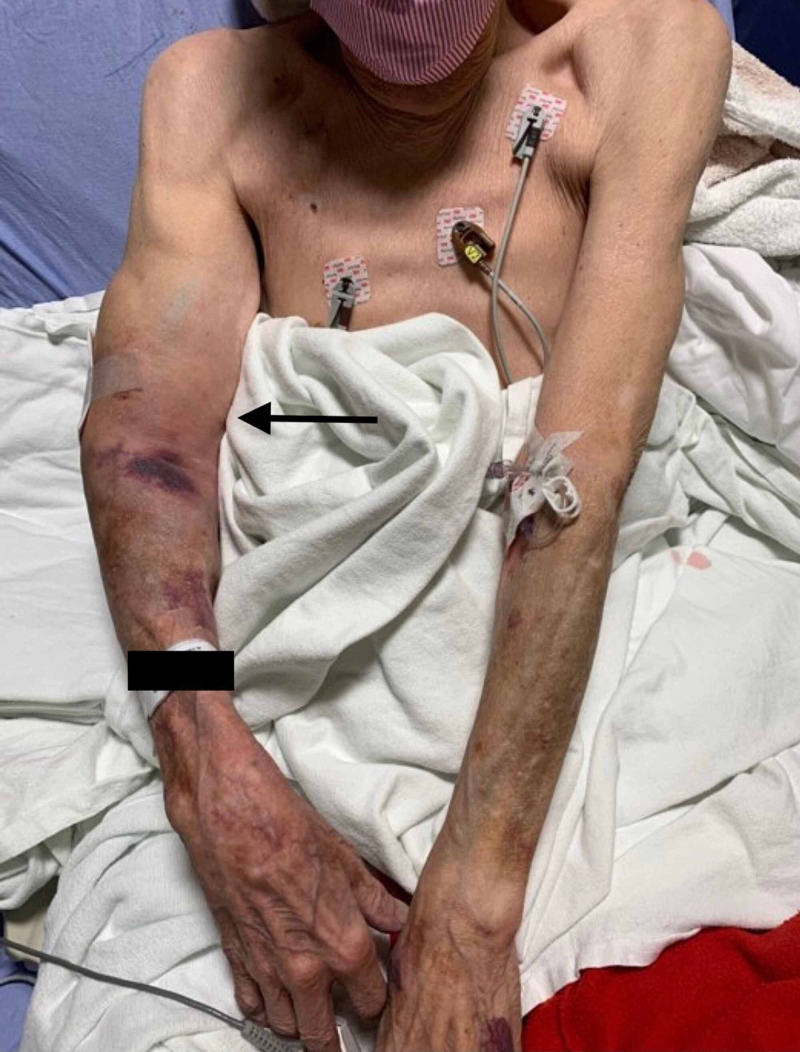
Right upper arm swelling

**Figure 2:**
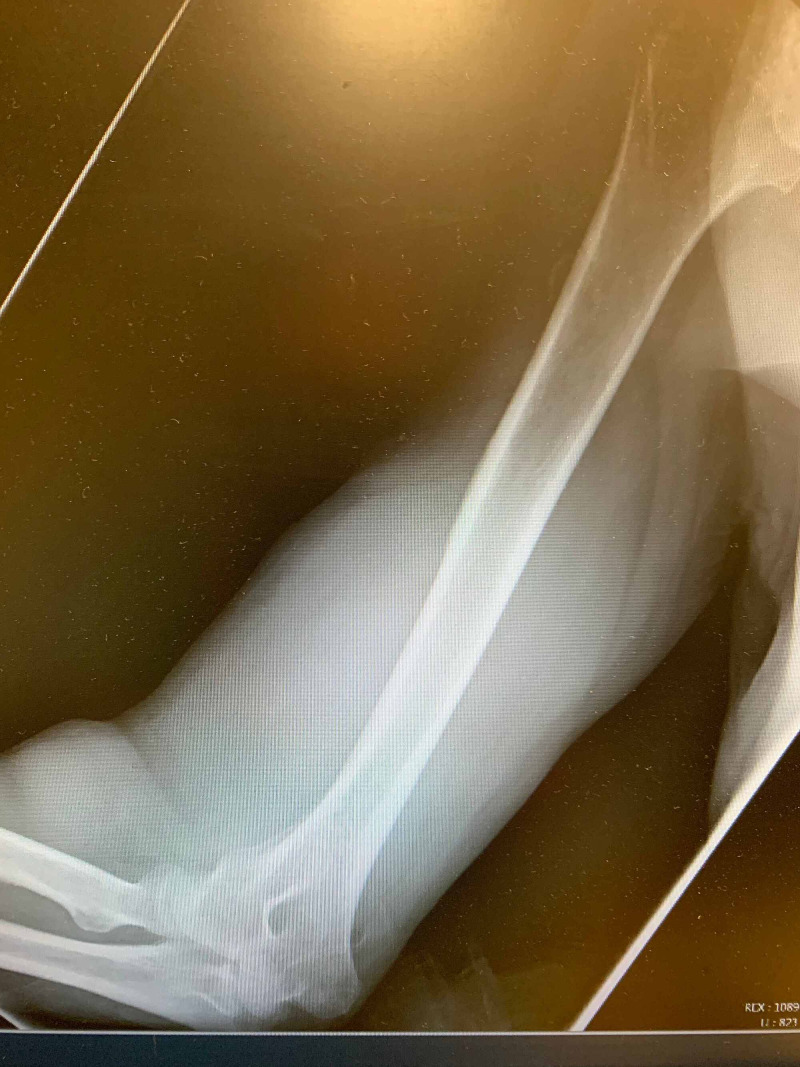
X-ray of the right upper extremity

**Figure 3:**
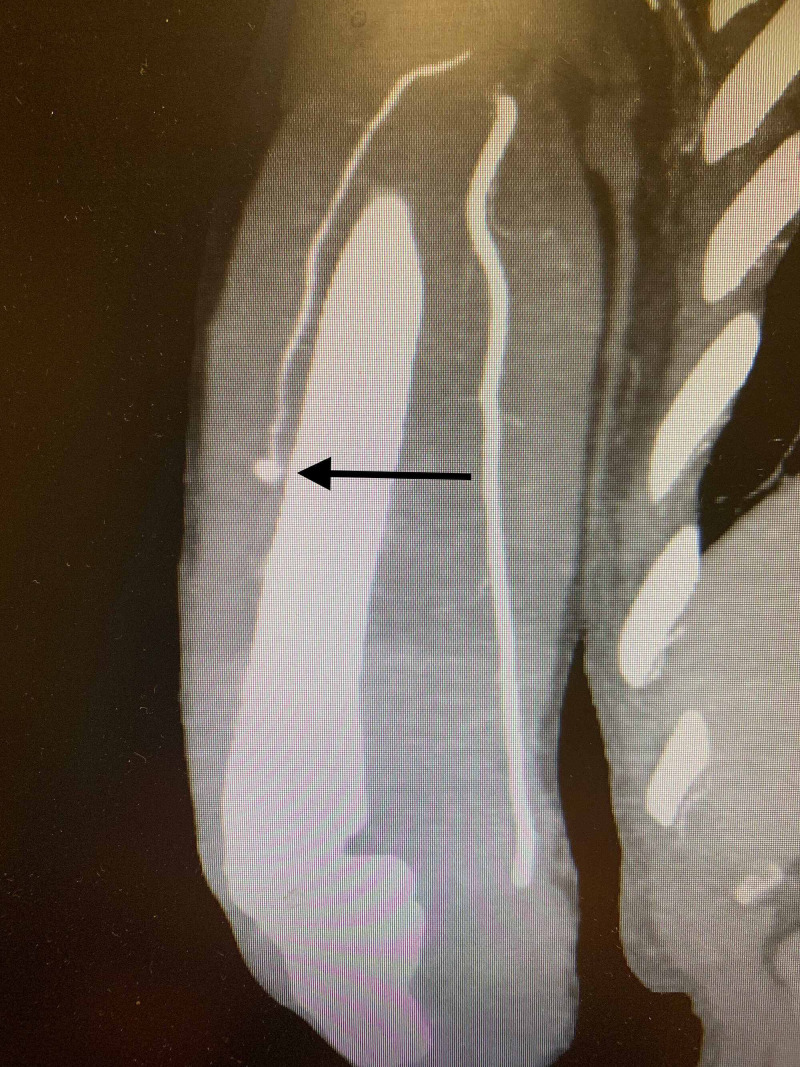
CTA of the right upper extremity demonstrating active bleed CTA: computed tomography angiogram

On reexamination, due to the patient complaining of increasing pain in his right arm, he had developed rapid progression of right arm swelling with associated pain of the upper arm and elbow (Figure 1). The upper arm had become significantly more edematous and firm to palpation. An X-ray of the right humerus and elbow were obtained but did not reveal an acute fracture (Figure 2). Due to the rapid progression of swelling and pain out of proportion to the exam, a CT angiogram (CTA) of the right upper extremity was ordered and orthopedic surgery was consulted for concerns of developing compartment syndrome. Orthopedic surgery evaluated the patient prior to CT and recommended completion of imaging with concern for possible vascular source but was in agreement with the suspected diagnosis of compartment syndrome based on the physical exam alone. During this period, the patient’s symptoms continued to progress with the development of numbness and tingling to the distal right upper extremity. CTA showed a hematoma within the right biceps musculature with evidence of active arterial extravasation from a branch vessel of the profunda brachii artery (Figure 3). The vascular surgery team was also consulted for input on the case given the concerns for an arterial bleed being the source for compartment syndrome. Ultimately, it was decided that the orthopedic surgery team would proceed to the operating room (OR) as intramuscular hematoma as a result of the arterial bleed was the likely source of compartment syndrome. Given the patients progressing physical exam findings and imaging, compartment pressures were not measured. Prior to leaving the ED, the patient had progressed to the point of having limited use of his right elbow, numbness and tingling of the distal right upper extremity, and was beginning to have difficulty with ROM with his right wrist.

The patient was taken for emergent fasciotomy of the right upper extremity by orthopedic surgery. Operatively, the profunda brachii artery was found to have pulsatile bleeding along the lateral septum of the anterior compartment after the compartment was released. The artery was clamped, tied, and cauterized, and a large hematoma was expressed deep to the anterior musculature. Next, the posterior fascia was incised with significant muscle expansion past the fascia but no muscle necrosis was identified. Following the compartment openings and bleeding control, the compartments were noted to be soft and further fasciotomy was not required. The patient maintained his distal pulses prior to surgery and noted significant improvement in pain following surgery. The patient continued to recover well from the procedure and was discharged on postoperative day two.

## Discussion

Classically, the teaching of compartment syndrome involves the six Ps: pain, pallor, poikilothermy, paresthesia, paralysis, pulselessness [3]. Pain out of proportion on physical exam and paresthesia should prompt concern for possible compartment syndrome whereas pulselessness is a late finding determined by compartment pressures exceeding arterial pressures [4]. Clinicians must have a high index of suspicion to promptly diagnose compartment syndrome in order for the patient to receive appropriate definitive care. While measuring compartment pressures may prompt consultants into action, normal measured compartment pressure alone does not rule out compartment syndrome [4]. Inter-rater reliability between examiners is variable, as seen in a study of physicians by Large et al. in which 30% of measurements resulted in a catastrophic error and few of the clinicians were able to measure all compartments of an extremity successfully [5]. Acute compartment syndrome not accompanied by fracture carries a higher risk in delayed diagnosis [3]. In this case, we present an atypical and delayed presentation of compartment syndrome following a fall caused by an arterial bleed without an associated fracture. This type of presentation is the vast minority of compartment syndrome cases, though it appears to be more prevalent in older, comorbid patients [6]. This case underscores the importance of serial examinations and a very high index of suspicion.

## Conclusions

Compartment syndrome carries high morbidity if not promptly diagnosed and treated. The emergency department physician plays a key role in identifying the early signs of compartment syndrome even when the clinical presentation and progression is atypical. Understanding the risks and key findings on exam ultimately allows for prompt surgical consultation and intervention.
